# *Withania somnifera* (Ashwagandha) Improves Fitness in Aged *Drosophila* by Reducing AKT Phosphorylation

**DOI:** 10.3390/ijms27188314

**Published:** 2026-09-18

**Authors:** Alexander D. Law, Mikah Brandes, Melissa Bollen, Amala Soumyanath, Doris Kretzschmar

**Affiliations:** 1 BENFRA Botanical Dietary Supplements Research Center, Oregon Health & Science University, Portland, OR 97239, USA; law@ohsu.edu (A.D.L.); soumyana@ohsu.edu (A.S.); 2 Oregon Institute of Occupational Health Sciences, Oregon Health & Science University, Portland, OR 97239, USA; 3 Department of Neurology, Oregon Health & Science University, Portland, OR 97239, USA

**Keywords:** Akt, *Drosophila melanogaster*, ashwagandha, locomotion, aging, longevity, NRF2, Keap, FOXO, insulin signaling

## Abstract

With increasing global life expectancy, there is a growing need for strategies to mitigate age-related declines in fitness and health. *Withania somnifera* (WS), commonly known as ashwagandha, has been traditionally recognized by ayurvedic medicine for its benefits in promoting healthy aging. More recently, it has also emerged as a potential treatment option for age-related diseases such as Alzheimer’s disease (AD), Parkison’s disease, and Huntington’s disease. WS has been shown to improve sleep quality, has adaptogenic and antioxidant properties, and enhances exercise performance by improving muscle strength and recovery. However, the molecular mechanisms underlying the effects of WS on aging are poorly understood. Therefore, we used *Drosophila melanogaster* flies to assess the impact of analytically characterized water (WSAq) extracts of WS root. To test effects during aging, the flies were treated at middle-age (4 weeks from eclosion) for two weeks. We found that WSAq reduced Akt phosphorylation in fly heads and that Akt is required to improve fitness in the fast phototaxis assays. We also determined that the effects of WSAq treatment depended on the Akt downstream pathways Tsc/mTOR, FOXO, and NRF. This supports that WS provides resilience to the age-related decline in fitness by modifying Akt signaling and affecting downstream pathways connected to aging and longevity.

## 1. Introduction

In the past two decades, advancements in healthcare, technology, and living conditions have led to a notable increase in human life expectancy. From 2000 to 2019, global life expectancy rose from 66.8 to 73.4 years according to the World health Organization (https://www.who.int/data/gho/data/themes/mortality-and-global-health-estimates/ghe-life-expectancy-and-healthy-life-expectancy, accessed on 14 May 2024). They forecast that by 2030, the global population of individuals aged 65 and older will exceed 1 billion, representing approximately one-eighth of the world’s population. This demographic shift brings with it challenges regarding age-related chronic diseases, particularly type 2 diabetes, arthritis, and neurodegenerative diseases like Alzheimer’s and Parkinson’s disease. The disparity between lifespan extension and healthspan, which has only increased by 5.4 years during the same period [1], shows the urgent need for healthy aging strategies, ideally not pharmacological treatments because while some may be effective in alleviating these diseases, they often come with adverse side effects and high costs.

Among various alternatives, dietary supplements, particularly herbal extracts, stand out as accessible and cost-effective candidates that can be used to promote healthy aging. *Withania somnifera* (L.) Dunal (WS), commonly known as ashwagandha, has been utilized in traditional medicine for centuries and is increasingly used in Western countries for its potential to ameliorate age-related conditions [2]. Decades of research have highlighted the health benefits of WS, with recent meta-analyses from several placebo-controlled human trials confirming positive outcomes. For instance, it has been reported that WS can significantly decrease stress and anxiety and significantly reduce serum cortisol levels [3,4], improve sleep in adults [5], promote muscle strength and physical performance [6], and ameliorate cognitive dysfunction and reduce inflammation [7]. Although these studies suggest broad benefits, they vary in their selection of study populations (including differences in age, gender, or patient groups), methods of preparing the WS extracts, and the actual phytochemical composition of the plant materials, making direct comparisons of efficacy difficult. In our current study, we utilized a highly inbred population of *Drosophila* and analytically characterized WS extracts to identify functional mechanisms of WS under maximally controlled conditions.

We have previously shown that a water extract of WS (WSAq) enhances physical fitness, while an ethanol extract (WSE) improves sleep in aging *Drosophila*. Additionally, we showed that both extracts alleviate stress-induced behavioral changes [8], with WSAq being more effective. However, the specific molecular mechanisms underlying these behavioral improvements in our model remain unexplored. Research in other systems has revealed that WS possesses neuroprotective and anti-tumorigenic properties (reviewed in [9,10]. The attributable bioactive compounds are found in both arial and root parts and include steroidal lactones, particularly withanolides such as withaferin A (WA) and withanone, alkaloids, flavonoids, phenolic acids, glycosides, saponins, tannins, and coumarins [11]. These compounds are known to affect signaling pathways related to cellular stress responses, apoptosis, and inflammation [11,12]. Notably, WA has been shown to induce apoptosis in cancer cells and reduce neuroinflammation in models of neurodegenerative diseases by modulating pathways including NF-κB, Pi3k/Akt, and MAPK [13]. Despite these findings, a detailed understanding of the molecular interactions and pathways influenced by WS in vivo remains limited.

The Akt signaling pathway is highly conserved and is involved in regulating cell growth, survival, metabolism, apoptosis, and longevity [14,15,16,17]. Akt signaling has also been found to be involved in aging and age-related diseases [18,19,20]. It is required for responses to oxidative stress and maintenance of protein homeostasis, which often become dysregulated with age. Accordingly, we used WS treatment and genetic manipulations in *Drosophila* to explore potential mechanisms related to Akt signaling, by which WS may contribute to resilience to age-related physical decline. The modulation of this pathway by WS therefore presents an opportunity to understand how WS might preserve cellular health.

## 2. Results

### 2.1. WSAq Decreases Phosphorylated Akt Without Changing Total Akt Protein Levels

**Figure 1:**
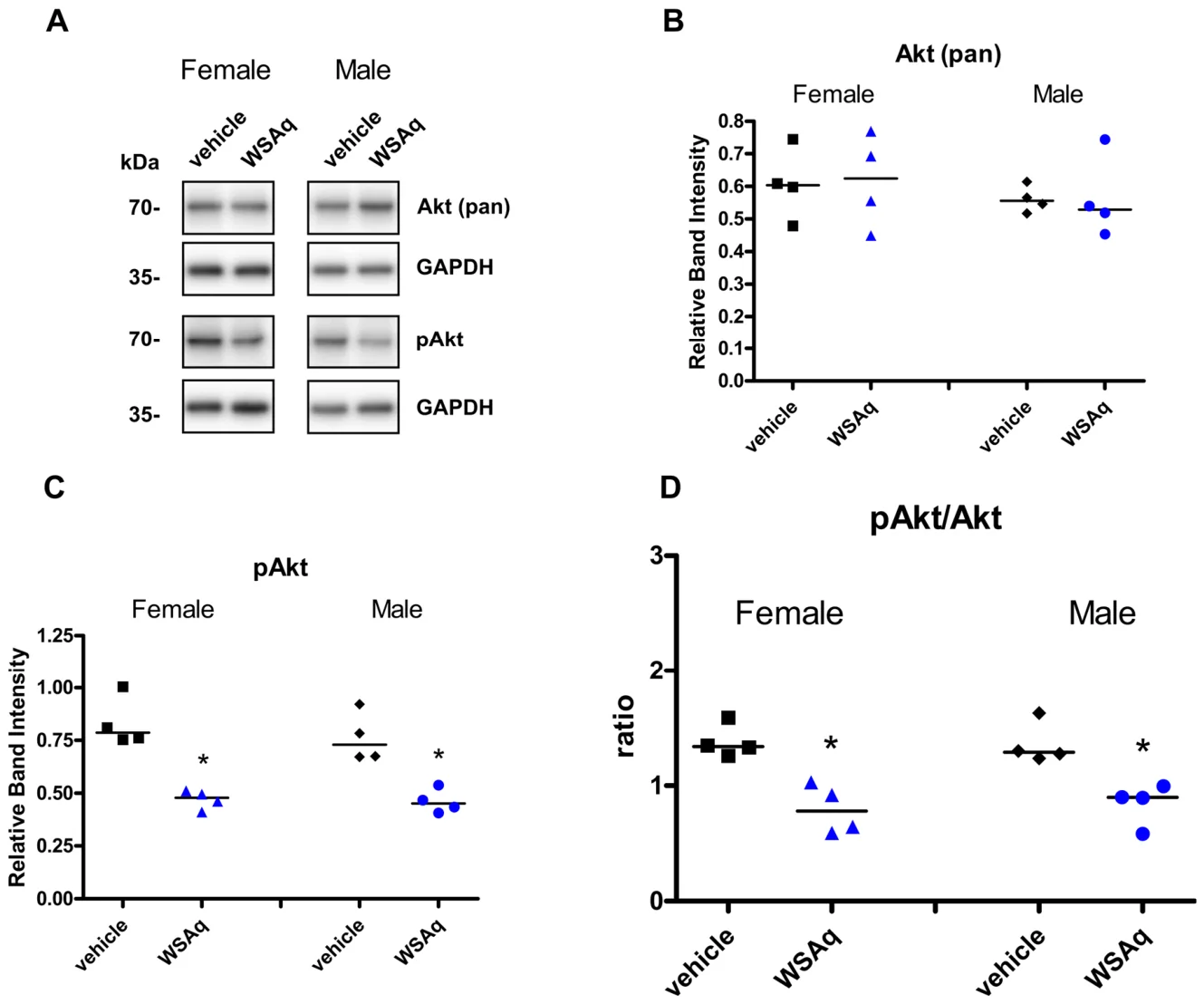
WSAq treatment reduces pAkt levels but not total Akt. (**A**) Representative Western blots. GAPDH was used as the loading control. (**B**) Total Akt levels are unchanged by WSAq while (**C**) pAkt levels are reduced with WSAq treatment compared to controls. (**D**) WSAq causes a reduction in pAkt levels relative to total Akt. Data are shown as individual points from 4 replicate Western blots with the median indicated. Statistical significance was assessed using a two-tailed Mann–Whitney test. *n* = 4, * *p* < 0.05 compared to vehicle. Original images are shown in Supplementary Figure S1.

As mentioned earlier, the Akt kinase (Akt) signaling pathway is a well-studied regulator of aging and longevity and since WS seems to provide resilience to aging, we tested whether it may act through Akt. To explore whether our WSAq extracts alter Akt levels, we aged CS flies to 28 days and treated them with our analytically characterized extracts for two weeks. We then performed Western blots of head lysates from both females and males using antibodies against both pan-Akt and pAkt. This revealed that the total Akt levels were not affected by WSAq treatment (Figure 1A,B); however, pAkt was significantly reduced in both females and males (Figure 1C). This is also evident when comparing the pAkt/Akt ratios (Figure 1D). This confirmed that WSAq does have an effect on Akt, reducing its phosphorylated form.

### 2.2. Akt Is Required for the Beneficial Effects of WSAq

To address the effect of WS on aging phenotypes in flies, we used fast phototaxis behavior, which is a standard measure of sensory and motor ability in flies. Furthermore, we previously showed that WSAq mitigated the age-related decline in phototaxis [8]. We therefore used phototaxis to test whether WSAq requires Akt for the improvement of this behavior during aging. To achieve this, we genetically increased or decreased Akt using the *elav*-GAL4 gene switch system (*elav*-GS). *elav*-GS is a mifepristone (RU486)-inducible, neuron-specific promoter construct that allows temporally and spatially controlled gene expression [21]. This allowed us to synchronize extract treatment with the onset of gene expression specifically in neurons. We focused on neuronal expression because our previous work, along with that of many others, has demonstrated the importance of the neuronal network in fast phototaxis behavior [22,23].

**Figure 2:**
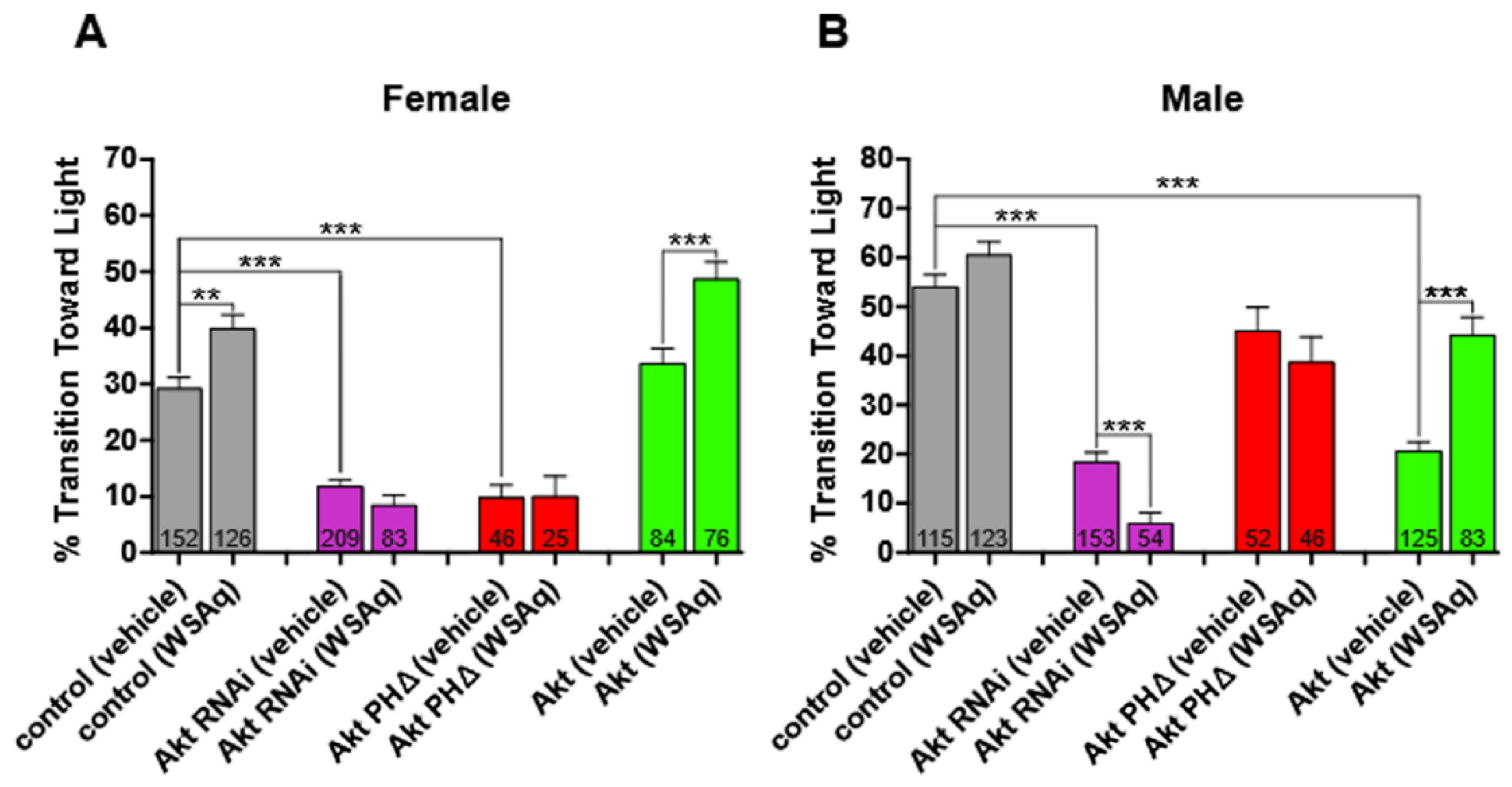
WSAq requires Akt for its effects. (**A**) Females and (**B**) males. WSAq treatment improves performance in control females but the effect is not significant in males. Knocking down Akt pan-neuronally or expressing the PHΔ mutation prevents the positive effects of WSAq. Overexpressing Akt increases the improvement by WSAq and also results in significant improvement in males. All flies carried the *elav*-GS construct and were 42 d old when tested. The number of flies used is given on the inside base of the bars. Data are shown as mean ± SEM for % transition toward light. Between-genotype comparisons: each vehicle-treated construct was compared to control (vehicle) using Kruskal–Wallis with Dunn’s multiple-comparisons post-test (WSAq groups excluded from this analysis). Between-treatment comparisons: vehicle vs. WSAq was compared within each construct using two-tailed Mann–Whitney tests. ***, *p* < 0.001; **, *p* < 0.01.

We first confirmed that the presence of *elav*-GS and the treatment with RU486 does not interfere with the beneficial effects of WSAq. To achieve this, we crossed flies carrying the *elav*-GS construct to the wildtype line CS so that the progeny carries only one copy of *elav*-GS, as do the experimental flies. Again, we aged the flies for the first four weeks of adulthood on standard food because we aimed to identify protective effects when treatment is started at middle-age. After that, we treated the flies for two weeks with either distilled water (vehicle) or WSAq at 0.5 mg/mL added to the food. All flies were treated with RU486. Similarly to what we previously showed, WSAq treatment improves phototaxis in female control flies (Figure 2A; gray bars). Also in agreement with the results in Holvoet et al. (2022) [8], we did not detect a significant improvement in males, though they performed slightly better when treated with WSAq (Figure 2B).

To address whether Akt is required for the effects of WSAq, we knocked down Akt using an RNAi construct. The Akt knockdown led to significant phototaxis defects in both sexes, and this was not improved by WSAq treatment (Figure 2A,B; purple bars). Treated males even performed significantly worse than the controls. We also expressed a pleckstrin homology (PH) domain deletion mutant of Akt (Akt PH^∆^). After initiation of the Akt signaling pathway by Pi3k92E, the PH^∆^ domain of Akt allows it to co-localize with the 3′-phosphoinositide-dependent kinase-1 (Pdk1) at membranes, thereby being phosphorylated and activated by phosphorylation via Pdk1 [24,25]. Expressing Akt PH^∆^ resulted in a phototaxis defect similar to the knockdown in females, which was also unchanged by WSAq treatment (Figure 2A; red bars). In males, expression of Akt PH^∆^ did not significantly reduce phototaxis but WSAq still had no effect (Figure 2B). We also overexpressed Akt with *elav*-GS. As shown in Figure 2A (green bars), treatment with WSAq again enhanced performance in females. In males, overexpressing Akt reduced phototaxis compared to vehicle control. However, now WSAq significantly improved performance (Figure 2B; green bars). Analyzing whether the genetic background interacted with the sex, we found that the overexpression of Akt had a significant sex-specific effect (Supplementary Table S1B). This interaction between sex and Akt overexpression could also be the cause for the significant sex-specific effects of WSAq treatment (Supplementary Table S1A). Together, the results show that the benefits on phototaxis induced by WSAq depend on Akt. That Akt PH^∆^, which is predicted to be much less phosphorylated than normal Akt, also prevents the effects of WSAq supports our results from Western blots that WSAq is acting via reducing Akt phosphorylation.

**Figure 3:**
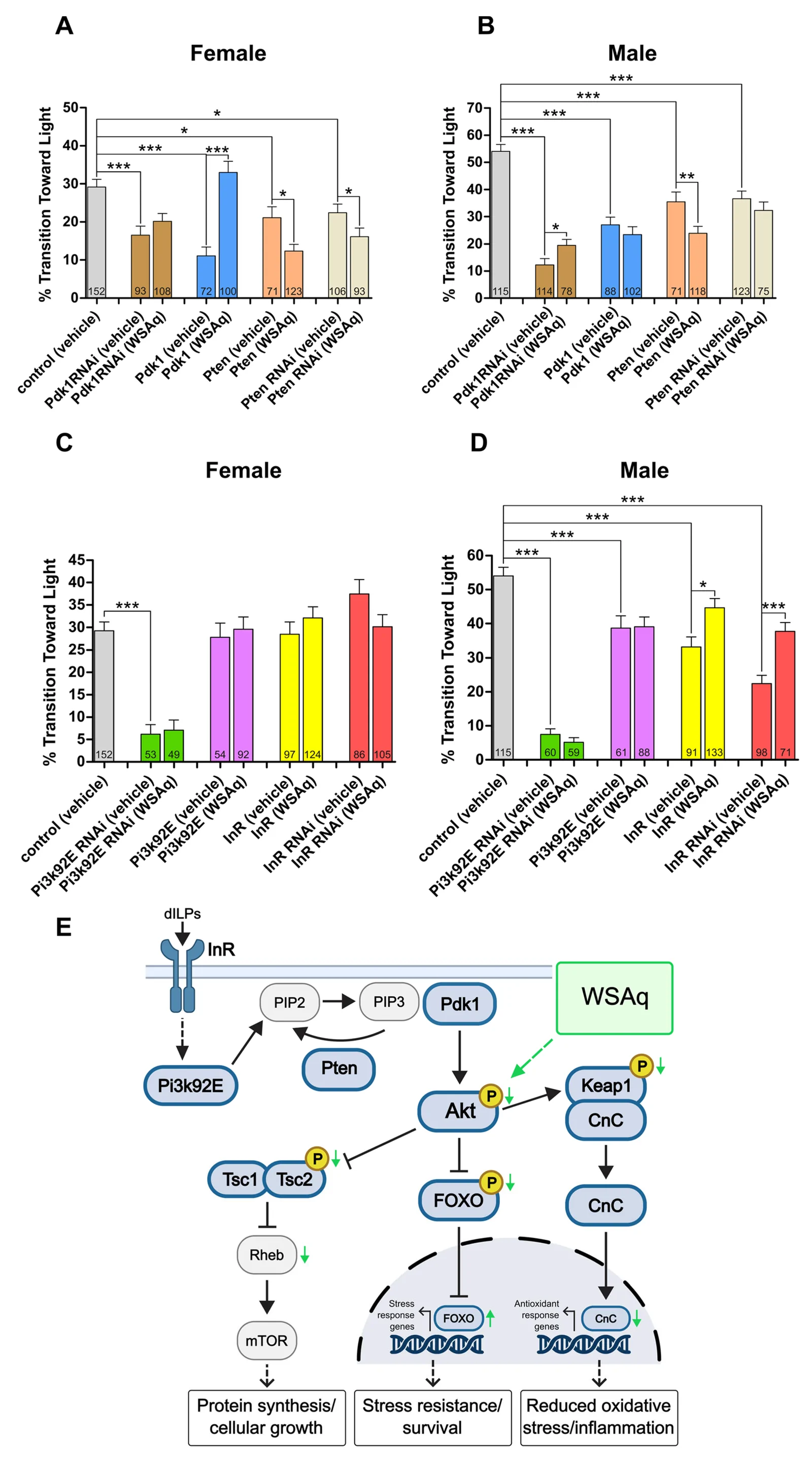
Adult neuron-specific manipulations of upstream Akt modulators. (**A**,**C**) Females and (**B**,**D**) males. (**A**,**B**). Knocking down the Pdk1 kinase prevents the improvement by WSAq whereas overexpression increases the WSAq effect. When overexpressing the Pten phosphatase, WSAq decreased the performance and this is also seen in flies with a knockdown of Pten. In males, WSAq had no effect on flies with a knockdown of Pten. (**C**,**D**) Manipulating Pi3k92E or InR prevented the effects of WSAq treatment in females. This is also seen in males for Pi3k92E but when manipulating InR, WSAq improved the performance in males. All flies contained *elav*-GS construct and were 42 d old when tested. The number of flies used is given on the inside base of the bars. Data are shown as mean ± SEM for % transition toward light. Between-genotype comparisons: each vehicle-treated construct was compared to control (vehicle) using Kruskal–Wallis with Dunn’s multiple-comparisons post-test (WSAq groups excluded from this analysis). Within-construct treatment comparisons: vehicle vs. WSAq was compared within each construct using two-tailed Mann–Whitney tests. ***, *p* < 0.001; **, *p* < 0.01; *, *p* < 0.05. (**E**) Schematic showing the insulin/Akt pathway. Upstream and downstream factors of Akt that have been tested for altering the effects of WSAq treatment are shown in blue. WSAq reduces the phosphorylation of Akt (small green arrow) which should result in decreased phosphorylation of its targets, thereby increasing Foxo nuclear translocation and reducing CnC release. Reduced Tsc2 phosphorylation disrupts the complex with Tsc1, decreasing its ability to interact and inhibit Rheb, which then activates mTOR (green arrows and lines).

To further test whether already reduced Akt phosphorylation does prevent the effects of WSAq, we tested flies with a knockdown of Pdk1. The knockdown resulted in decreased phototaxis in both sexes in untreated flies and it prevented the beneficial effect of WSAq in females and only induced a small positive effect in males (Figure 3A,B; brown bars). Overexpression of Pdk1 also had a negative effect on phototaxis in the untreated controls; however, WSAq treatment strongly improved phototaxis to the normal levels in controls in females (blue bars). In males, WSAq treatment had no effect and this sex-specific effect of WSAq was significant (Supplementary Table S1A). In this case the overexpression of Pdk1 alone did not induce a sex-specific effect (Supplementary Table S1B). We also manipulated the expression of the phosphatase and tensin homolog (Pten). Pten reduces Akt phosphorylation by dephosphorylating PIP3 to PIP2 which prevents the PIP3-dependent recruitment of Pdk1 to the membrane and, consequently, Akt phosphorylation [26]. Overexpression of Pten reduced the performance in the untreated flies and this was aggravated by WSAq treatment (Figure 3A,B; pale orange bars). Knocking down Pten also reduced phototaxis and unexpectedly WSAq treatment did not ameliorate this and even made it slightly worse in females (tan bars). Nevertheless, these experiments confirmed that manipulations that are aimed to decrease Akt phosphorylation, Pdk1 knockdown and Pten overexpression prevented the positive effects of WSAq. Furthermore, WSAq restored normal phototaxis in Pdk1 overexpressing females, suggesting that it can reduce the negative effect of increased phosphorylation of Akt.

Next, we turned our attention to factors upstream of Pdk1 by overexpressing Pi3k92E (also known as p110D) and knocking it down using RNAi (Figure 3C,D). Pi3k92E encodes the primary class I phosphoinositide 3-kinase in *Drosophila* and generates PIP3 from PIP2, thereby promoting the co-localization of Pdk1 and Akt at the membrane and Akt phosphorylation (for a review, see Manning and Toker, 2017, [14,27]). Knocking down Pi3k92E led to a severe phototaxis defect in both females and males, with no effect from WSAq treatment (Figure 3C,D; green bars). This is in agreement with our results of the Pdk1 knockdown, further supporting that WSAq requires Pdk1 and Akt phosphorylation for its effect. Pi3k92E overexpression also resulted in WSAq treatment having no effect (Figure 3C,D; pink bars), suggesting that it might not be sufficient to increase Pdk1 activity.

In addition, we overexpressed the *Drosophila* insulin-like receptor (InR) and knocked it down using RNAi (Figure 3C,D). InR encodes a single insulin/IGF-like receptor that binds multiple insulin-like peptides, thereby activating Pi3k-Akt signaling cascades that regulate cell and organ growth, nutrient-dependent body size, and metabolic homeostasis [28,29]. When overexpressing InR, WSAq could still improve the phototaxis, but this only reached significance in males (Figure 3D; yellow bars). This suggests that at least in males, WSAq can reduce the overactivation of the Akt pathway by reducing Akt phosphorylation. The knockdown of InR prevented the effects of WSAq in females, even trending towards reducing phototaxis (Figure 3D,C; yellow bars). While this is in agreement that InR-induced activation of Akt is required for the beneficial function of WSAq, the InR knockdown in males surprisingly increased the effects of WSAq (Figure 3D; red bars). Supplementary Table S1A shows that this sex-specific effect is significant and so is the interaction between the knockdown and sex (Supplementary Table S1B).

**Figure 4:**
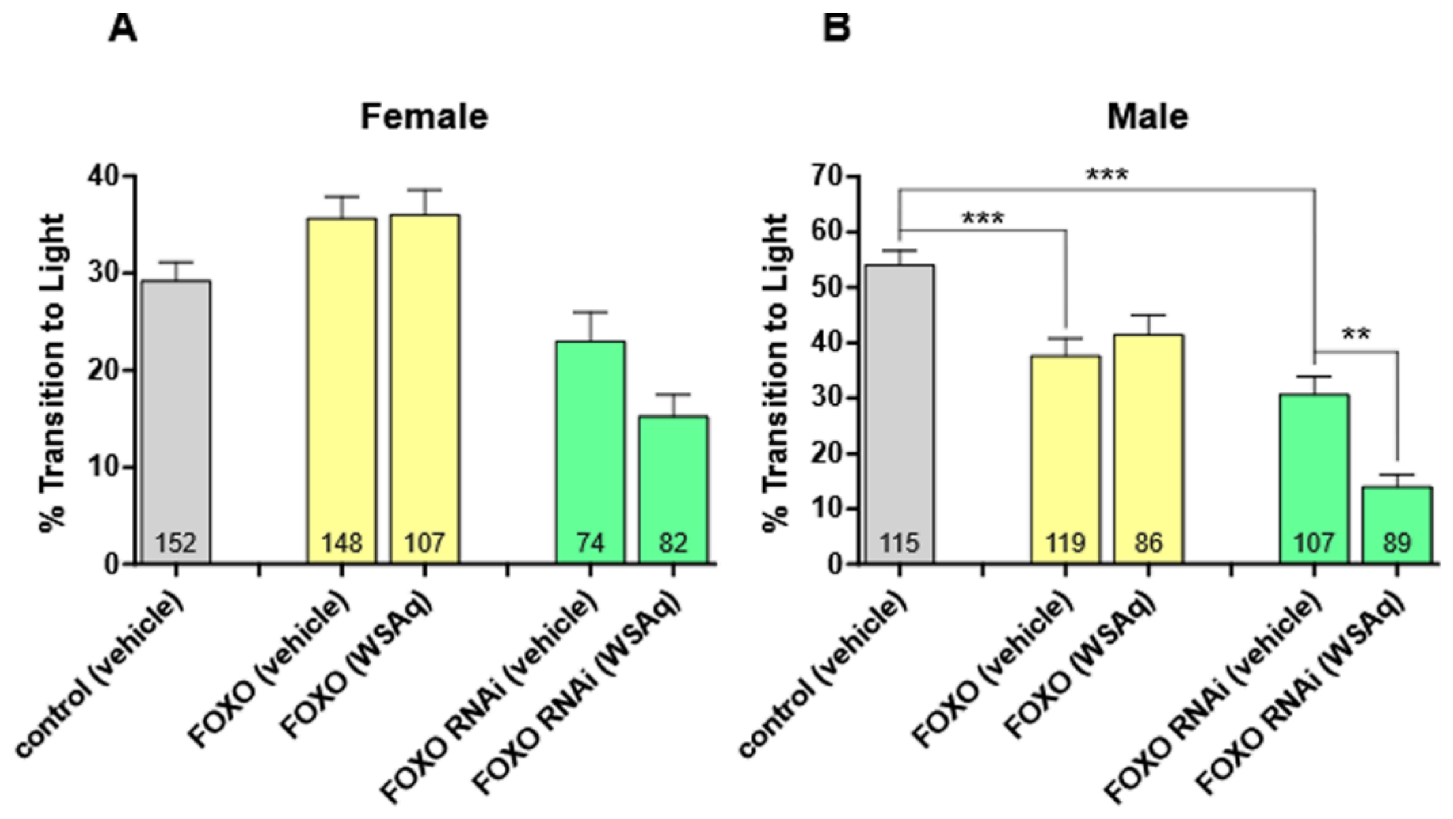
Foxo manipulations prevent the protective effects of WSAq. (**A**) Females and (**B**) males. WSAq had no effect in flies overexpressing Foxo. WSAq treatment decreased the performance in Foxo knockdown flies but only reached significance in males. All flies contained *elav*-GS construct and were 42 d old when tested. The number of flies used is given on the inside base of the bars. Data are shown as mean ± SEM for % transition toward light. Between-genotype comparisons: each vehicle-treated construct was compared to control (vehicle) using Kruskal–Wallis with Dunn’s multiple-comparisons post-test (WSAq groups excluded from this analysis). Between-treatment comparisons: vehicle vs. WSAq was compared within each construct using two-tailed Mann–Whitney tests. ***, *p* < 0.001; **, *p* < 0.01.

We then assessed the impact of WSAq on downstream targets of Akt. We first focused on the forkhead box sub-group O (Foxo) transcription factors, of which *Drosophila* has just one [30]. Phosphorylated and therefore activated Akt inhibits Foxo by phosphorylation, which excludes it from the nucleus where it normally regulates the expression of genes involved in metabolism, stress resistance, proliferation, and apoptosis [31,32]. Under conditions of cellular stress or reduced levels of growth factors, Foxo is activated to maintain cellular energy homeostasis [30]. Foxo was also of interest due to its connection to aging and longevity, ranging from species like *C. elegans* to humans [32,33,34]. This includes experiments in *Drosophila*, showing that overexpression of Foxo increases lifespan [35,36]. In our experiments, overexpressing Foxo in females improved phototaxis compared to the control, although this was not significant. WSAq treatment had no additional effect (Figure 4A; pale yellow bars). Males expressing additional Foxo performed worse than the control and WSAq supplementation also did not induce a change (Figure 4B; pale yellow bars). Inducing the Foxo RNAi construct in neurons reduced phototaxis performance in both sexes and this was not improved by WSAq in females, but it further reduced the performance in males (Figure 4A,B; pale green bars). That WSAq could not improve phototaxis in the Foxo knockdown but even aggravated it in males was unexpected. However, Foxo can also activate Akt. Reducing Akt activation by WSAq in addition to reducing Foxo could result in a severe reduction in Akt activity that has negative effects. This is in agreement with our result in Figure 2, showing that a reduction in Akt, even in controls, reduces phototaxis.

**Figure 5:**
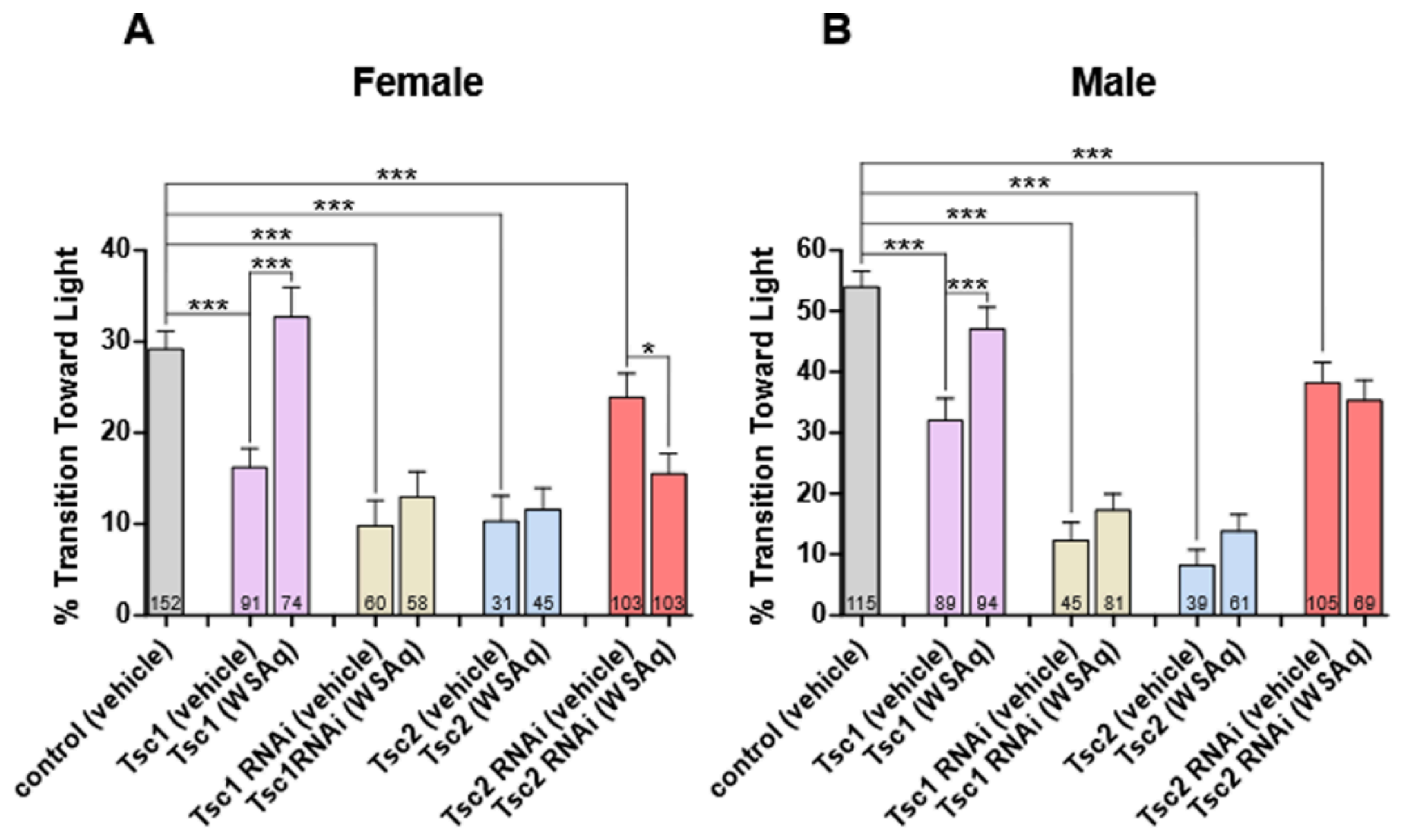
The Tsc/mTOR pathway is required for the improvement of phototaxis by WSAq. (**A**) Females and (**B**) males. Overexpression of Tsc1 increased the positive effects of WSAq, also reaching significance in males. The knockdown of Tsc1 or Tsc2 prevented the effects of WSAq. All flies contained *elav*-GS construct and were 42 d old when tested. The number of flies used is given on the inside base of the bars. Data are shown as mean ± SEM for % transition toward light. Between-genotype comparisons: each vehicle-treated construct was compared to control (vehicle) using Kruskal–Wallis with Dunn’s multiple-comparisons post-test (WSAq groups excluded from this analysis). Between-treatment comparisons: vehicle vs. WSAq was compared within each construct using two-tailed Mann–Whitney tests. ***, *p* < 0.001; *, *p* < 0.05.

Another downstream pathway regulated by Akt is the mechanistic target of rapamycin (mTOR) signaling. Akt does not regulate mTor directly, rather activated Akt inhibits the function of the tuberous sclerosis complex 1 and 2 (Tsc1/2). When Tsc2 is phosphorylated by Akt, the Tsc1/2 complex is disrupted, preventing its inhibition of Ras homolog enriched in brain (Rheb), which is a GTPase that promotes the activation of mTOR [37]. mTOR signaling has been shown to play an important role in aging [38,39] and overexpressing Tsc1 in mice or *Drosophila* has been shown to increase lifespan [40,41]. When we overexpressed Tsc1, we saw a reduction in phototaxis, which was significantly improved in both females and males with WSAq treatment, reaching similar levels as in the controls (Figure 5A,B; pink bars). Although both sexes showed an improvement in phototaxis, the effect of WSAq was significantly stronger in females (Supplementary Table S1A). Overexpression of Tsc1 alone did not have a sex-specific effect (Supplementary Table S1B). Overexpression of Tsc2 also caused a defect, albeit much worse than Tsc1, which WSAq was unable to ameliorate in either females or males (Figure 5A,B; tan bars). Knocking down Tsc1 or Tsc2 also decreased performance in the phototaxis tests, with WSAq treatment aggravating it in females. Together, these findings suggest that the behavioral benefits of WSAq require the presence of the Tsc1/Tsc2 complex due to WSAq not being protective in the knockdowns or even worsening the phenotype.

**Figure 6:**
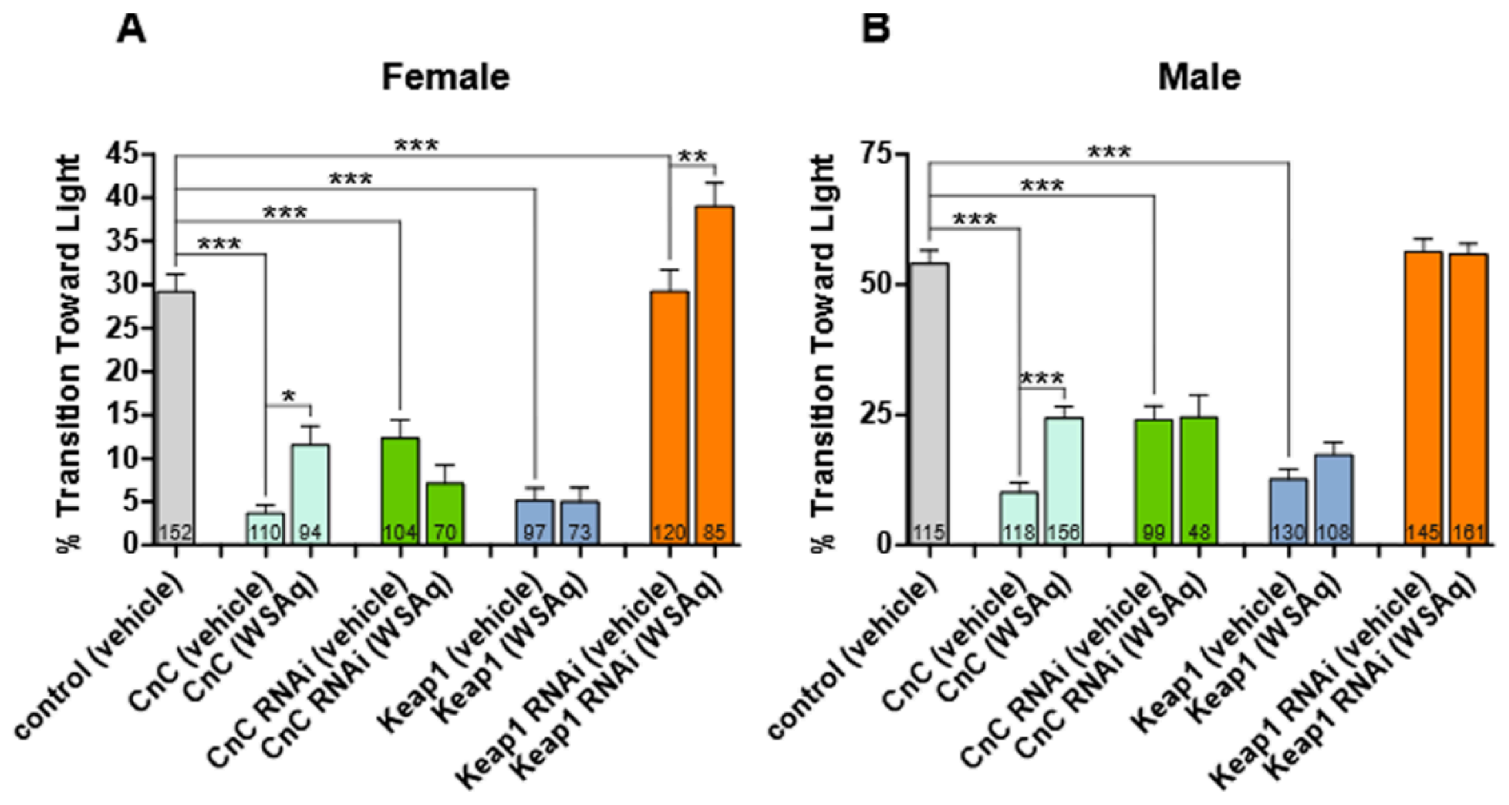
Improvement of phototaxis by WSAq requires the CnC/Keap 1 pathway. (**A**) Females and (**B**) males. WSAq still increases the performance when CnC is overexpressed or Keap1 knocked down (with the exception of the Keap1 knockdown in males). Knocking down CnC or overexpressing Keap1 prevented the positive effect of WSAq. All flies contained *elav*-GS construct and were 42 d old when tested. The number of flies used is given on the inside base of the bars. Data are shown as mean ± SEM for % transition toward light. Between-genotype comparisons: each vehicle-treated construct was compared to control (vehicle) using Kruskal–Wallis with Dunn’s multiple-comparisons post-test (WSAq groups excluded from this analysis). Within-construct treatment comparisons: vehicle vs. WSAq was compared within each construct using two-tailed Mann–Whitney tests. ***, *p* < 0.001; **, *p* < 0.01; *, *p* < 0.05.

The last target we addressed was nuclear factor erythroid 2-related factor 2 (Nrf2), which is a key factor in oxidative stress responses [42,43]. Akt can regulate the phosphorylation of Kelch-like ECH-associated protein 1 (Keap1) either indirectly through its effects on GSK3β or directly by phosphorylating it under certain stress conditions [42,44,45,46]. This results in the release of Nrf2 and its translocation to the nucleus where it activates the transcription of antioxidant genes. WS extracts have previously been reported to exhibit antioxidant properties in both in vitro and in vivo models [47,48,49,50,51,52]. Therefore, we tested whether the beneficial effects of WSAq on phototaxis are mediated by its role in regulating the cap ‘n’ collar (CnC, the fly Nrf2)/Keap1 pathway, which regulators oxidative stress response in flies analogous to this pathway in mammals. Overexpressing CnC pan-neuronally impaired phototaxis compared to controls in females and males, which was significantly improved by WSAq treatment (Figure 6A,B; pale turquoise bars). Although knocking down CnC also resulted in a phototaxis impairment, this was not improved by WSAq treatment in either gender (Figure 6A,B; green bars). The opposite was true for the manipulation of Keap1. Overexpression of Keap1 also caused reduced phototaxis compared to controls, but WSAq could not ameliorate this in either females or males (Figure 6A,B; blue-gray bars). The Keap1 knockdown had no significant effect when compared to controls but was improved with WSAq treatment in females (Figure 6A; orange bars). Supplementary Table S1A,B show that the interaction between treatment and sex is significant whereas the interaction between the Keap1 knockdown and sex is not.

While increasing NRF2 activity can be beneficial and result in lifespan extension, mainly under conditions of higher oxidative stress, a consistent increase in Nrf2 has been shown to induce senescence [53,54]. Furthermore, expressing high levels of CnC in *Drosophila* reduces lifespan [55]. This is in agreement with our findings that CnC overexpression aggravated the phototaxis defect in the aged flies. That CnC overexpression was significantly improved by WSAq suggests that the decreased Akt phosphorylation, directly or indirectly prevents the phosphorylation of Keap1 and therefore the release of active CnC. That WSAq had no effect in the CnC knockdown or Keap1 overexpression also supports that the effects of WSAq are mediated by altering the CnC/Keap1 pathway.

## 3. Discussion

As global life expectancy continues to rise, there is great interest in promoting healthy aging and mitigating age-related declines in fitness and health. *Withania somnifera* (WS) has long been used in traditional medicine for its potential benefits in promoting healthy aging and is now also increasingly popular in Western societies. It has been shown to improve performance, muscle strength and neuromuscular coordination in athletes and the general population [6,56,57]. In previous studies, we found that WS also improved the fitness and locomotion in aged *Drosophila* (Holvoet, 2022 [8]). We therefore used this well-characterized model to explore the mechanisms and genetic pathways that mediate this beneficial function of WS. *Drosophila* allowed us to tightly control the experimental conditions and, due to the availability of a plethora of overexpression and knockdown lines, address the role of specific genes in mediating the effects of WS.

We showed that WS treatment significantly decreases the levels of phosphorylated Akt (pAkt) without altering total Akt protein levels. We then confirmed that Akt is required for mediating the effects of WSAq because a knockdown of Akt prevented the positive effects of WSAq. However, it is possible that WSAq could not improve the behavior because the knockdown reduced the phototaxis to such a degree that WSAq could not overcome this. We think this is unlikely because in other cases where the genetic manipulations caused a severe phototaxis defect, WSAq could still improve the behavior (for example in the CnC overexpression). Furthermore, WSAq improved phototaxis in Akt overexpressing flies, also supporting the role of Akt in mediating the effects of WSAq. This was especially obvious in males in which overexpression of Akt reduced performance compared to controls and WSAq treatment prevented this effect, presumably by inhibiting the negative effects of hyperactive Akt signaling. This effect of WSAq treatment on males was significantly stronger than in females. Genetic manipulations that reduced Akt phosphorylation confirmed that WSAq acts on pAkt, as PDK1 knockdown or PTEN overexpression prevented the beneficial effects of WSAq. While Akt signaling has mostly been associated with promoting cell growth and survival, hyperactivation of Akt can trigger cell death as a protective mechanism in cancer [58]. In agreement with this, treatment with WS (or its constituent withaferin A) have been described to have positive effects in several cancer models. This was originally observed in a macrophage cell line established from a tumor of a mouse with Abelson murine leukemia virus (Oh, 2008 [59]). It was found that WA inhibits inflammation by suppressing nitric oxide production and inducible nitric oxide synthase (iNOS) expression through the inhibition of Akt phosphorylation and the subsequent down-regulation of NF-κB activity in these cells when stimulated with lipopolysaccharide (LPS) (Oh, 2008 [59]). Reduced phosphorylated Akt (pAkt) has also been observed in a mouse prostate cancer (TRAMP) model (Suman, 2016 [60]), human pulmonary epithelial cells (Oh, 2009 & Cai, 2014 [61,62]), and glioblastoma cells (Grogan, 2013 [63]).

Increased activation of Akt signaling has been found to promote neurodegenerative diseases. Akt hyperactivation has been connected with increasing inflammation, Tau hyperphosphorylation, Aβ deposition, cognition impairment, and synaptic damage, thereby promoting Alzheimer’s disease [64]. In agreement with these findings, knocking down Akt in transgenic *Drosophila* expressing human Aβ ameliorated learning deficits and extended lifespan [65]. Furthermore, reducing Akt signaling improved age-related phenotypes in the wildtype background by increasing locomotion, reducing cell death, and increasing lifespan [66]. Chen and colleagues also found that pAkt levels are increased with aging whereas total Akt levels are not affected by age and an increase in pAkt was also found in some tissues in aged mice and in the nervous system of rats [67]. Lastly, caloric restriction and reduced insulin signaling, which inhibit Akt activity, have been connected to increasing lifespan and healthy aging in many animal models and possibly also in humans [68]. That upstream activation of Akt also plays a role in the effects of WSAq was confirmed in our experiments where manipulating insulin/PI3k mostly prevented the effects of WSAq.

Lastly, we investigated which downstream pathways of Akt are affected by WSAq treatment. We tested genetic interactions with genes in three pathways; the TSC/TOR pathway, the FOXO pathway, and the Nrf2 (CnC) pathway. Knockdowns of genes in all three pathways prevented phototaxis improvement by WSAq, suggesting that reduced activation of Akt interferes with all three downstream pathways. However, the downstream network of Akt targets is quite complex and we also could not test some pathways due to knockdowns inducing lethality and/or strongly affecting locomotion already in young flies (for example, GSK3β). As described above, the FOXO transcription factor can activate genes that protect against several cellular stresses and it has been shown to promote longevity [33,34,35,36]. FOXO is inhibited by Akt and due to WSAq reducing Akt phosphorylation and activation, we hypothesize that it promotes protective mechanisms mediated by FOXO. Similarly, the Tsc/TOR pathway has been shown to regulate aging and increasing Tsc1 in mice or *Drosophila* has been shown to increase lifespan [40,41]. Like FOXO, Tsc is inhibited by active Akt and therefore WSAq treatment would increase activity of the Tsc/TOR pathway. Although reduced Akt phosphorylation should reduce the phosphorylation of its targets, we could not confirm this by Western blots because the antibodies to test this are not available for *Drosophila*.

The last pathway we investigated is Nrf2/Keap1 signaling, which is a key regulator of oxidative stress responses. Since Nrf2/CnC upregulates the expression of antioxidant genes, it may seem counterintuitive that the overexpression of CnC results in quite severe phototaxis defects in the wildtype background. However, this result is in agreement with published studies that overexpression of CnC in *Drosophila* reduces lifespan [55]. This has been confirmed by another group which showed that overexpression or sustained activation of Nrf2/CnC accelerated aging by deregulating insulin/insulin-like growth factor signaling (IIS), leading to hyperglycemia, and exhaustion of energy stores (Gumeni, 2023 [69]). This study also showed that knocking down one of the eight insulin-like peptides in flies overexpressing CnC suppressed premature aging. Akt activates Nrf2/CnC by phosphorylating Keap1, allowing Nrf2/CnC to translocate to the nucleus [70]. Consequently, reducing Akt activity by WSAq would maintain Nrf2/CnC in the complex with Keap1 and reduce NRF activity, thereby ameliorating the negative effects of CnC overexpression on phototaxis performance.

Interestingly, we found some sex-specific effects of WSAq but even more so interactions between sex and the genetic background caused by our knockdown and overexpression manipulations. However, differences in the insulin/Akt signaling pathway and its components between males and females have been described previously. This includes higher levels of InR and Pdk1 in females [71] and different consequences of altering signaling by reducing the levels of the InR in males versus females [72]. We therefore assume that altering the expression levels of downstream factors of InR by our genetic manipulation resulted in the different behavioral changes in males than females shown in Supplementary Figure S1B. These analyses also showed significant interactions between sex and WSAq treatment for flies with Akt overexpression, Pdk1 overexpression, InR knockdown and Tsc1 overexpression. In the case of the InR knockdown and Akt overexpression, we also found a significant interaction between sex and genetic background. As described by Graze et al. [72] the InR knockdown shows different transcriptomic changes in males and females which may cause the different responses to WSAq treatment. Similarly, the interaction of sex and the genomic background could underly the sex-specific response to WSAq in the case of Akt overexpression. In contrast, the sex-specific response to WSAq does not correlate with a significant interaction between sex and genetic background when Pdk1 or Tsc1 is overexpressed. In the case of Pdk1 overexpression, this could be related to higher levels of Pdk1 in females [71]. WSAq could be more effective at reducing pAkt in females in which its levels are already higher due to the combined effects of increased Pdk1 levels and its overexpression. However, future experiments are needed to address these sex-specific effects of WSAq.

## 4. Materials and Methods

### 4.1. Fly Stocks

Experiments without genetic manipulation were performed with *Drosophila melanogaster* wildtype Canton-Special strain (CS), originally provided by Martin Heisenberg (University Würzburg). All UAS stocks were obtained from Bloomington and included the following lines:

UAS-Akt (BDSC #8191); UAS-Akt RNAi (BDSC #31701); UAS-Akt PH^∆^ (BDSC #80935); UAS-InR (BDSC #8262); UAS-InR RNAi (BDSC #51518); UAS-Pi3k92E (BDSC #8287); UAS-Pi3k92E RNAi (BDSC #35798); UAS-Pdk1 (BDSC #19690); UAS-Pdk1 RNAi (BDSC #34936); UAS-Pten (BDSC #82170); UAS-Pten RNAi (BDSC #25841); UAS-FOXO (BDSC #80946); UAS-FOXO RNAi (BDSC #32427); UAS-Tsc1 (BDSC #80963); UAS-Tsc2 (BDSC #91239); UAS-Tsc1, Tsc2 (BDSC #80576); UAS-Tsc1 RNAi (BDSC #35144); UAS-CnC (BDSC #25984); UAS-CnC RNAi (BDSC #32407); UAS-Keap1 (BDSC #15427); UAS-Keap1 RNAi (BDSC #40932).

For all experiments, *elav*-GAL4 GeneSwitch virgin females were crossed to UAS-carrying males. The *elav*-GAL4 GeneSwitch line was a gift from Jadwiga Giebultowicz. Flies were maintained on standard Drosophila food at 24 °C under a 12:12 h light–dark cycle.

### 4.2. Plant Material

*W. somnifera* plants were grown at Oregon’s Wild Harvest (OWH), Redmond, OR, USA. The roots were harvested (OWH lot number 201000162) and voucher samples were deposited in the Oregon State University (OSU) Herbarium (voucher number OSC-V-265405) and in our laboratories (Code number BEN-WS-8) at Oregon Health & Science University (OHSU). Aqueous extracts were prepared as described previously [8]. Briefly, the dried roots were boiled in deionized water under reflux for 90 min, the mixture was filtered through a kitchen sieve to remove larger plant particles, and the extracts were centrifuged at 3750 rpm for 10 min. The supernatant was then filtered through Whatman filter paper (Grade 1, 90 mm) and the extract frozen and lyophilized into a powder. Extracts were given a lot number and stored at −20 °C until use. In this study, we used WSAq 10 and WSAq 20. Voucher samples of the extracts are stored in our laboratories under these lot numbers. The extracts were analyzed for their withanolide content by liquid chromatography coupled with mass spectrometry (LC-MS) [8].

### 4.3. Aging and WS Administration of Flies

Newly eclosed flies were collected every other day under light CO_2_ anesthesia, transferred to standard food, and aged to four weeks. At four weeks old, flies were transferred to standard food containing WSAq at a concentration of 0.5 mg/mL, prepared by diluting a WSAq 5 mg/mL stock aqueous solution into the food. Control flies were transferred to food with equal amounts of the vehicle (water 10%) added. Flies were aged for an additional two weeks, with fresh control or supplemented food being provided after one week. Consumption of the control and supplemented food at equal rates was confirmed previously [8]. We also confirmed that the withanolide content of supplemented food was stable for the one week it was used as measured by LC-MS [8]. For genetic experiments, in addition to the WSAq extract being added to the food, RU486 (M8046, Sigma-Aldrich, St. Louis, MO, USA) was added at a final concentration of 20 µM to induce gene construct expression. Males and females were aged together then tested and analyzed separately.

### 4.4. Phototaxis Assay

Fast phototaxis assays were conducted in the dark as previously described in [10,73] using the countercurrent apparatus described by [74] and a single light source. A detailed description of the experimental conditions can be found in [75]. Briefly, flies were transferred to the apparatus in groups of 10–15 flies, shaken to the bottom of the tube and allowed to transition toward the light in 5 consecutive runs, each lasting 6 s. Flies were then scored based on the tube they were contained in at the end of the final run (staying in tube 1 was scored as 0%, making the transitions every time was scored 100%, with intermediate percentages in the other tubes). Statistical analyses comparing vehicle and WSAq-treated groups were performed with GraphPad Prism v5 using nonparametric Mann–Whitney tests due to the values not being normally distributed (determined with the D’Agostino and Pearson omnibus test). To determine the effect of construct expression without WSAq treatment, Kruskal–Wallis tests with Dunn’s multiple comparison post-test were used due to multiple groups being compared. To determine sex-specific effects, the values of flies with an altered genetic background where normalized to the control flies and the difference determined as % change. In the case of WSAq, the values of treated flies were normalized to the flies receiving only the vehicle. Statistical analyses was then done using two-way ANOVA in GraphPad Prism v5.

### 4.5. Western Blots

To detect Akt and phospho-Akt (pAkt), 15 adult fly heads were dissected on an ice-cold plate, homogenized in 80 μL of 1.25 × LDS Sample Buffer (B0008, ThermoFisher, Waltham, MA, USA) supplemented with 50 mM tris(2-carboxyethyl)phosphine (TCEP) as a reducing agent along with protease and phosphatase inhibitors (5872S, Cell Signaling Technology, Danvers, MA, USA), and centrifuged at 10,000× *g* for 10 min at 4 °C. The supernatant was heated to 70 °C for 10 min to denature proteins. The equivalent of about two heads was electrophoresed through 8% bis-tris gels (ThermoFisher NW0082) to achieve separation of proteins, which were then transferred to PVDF membranes (ISEQ85R, MilliporeSigma, Burlington, MA, USA). Membranes were blocked with 5% BSA dissolved in 1 × TBST (Tris-buffered saline + 0.1% TWEEN-20) and then probed with primary and secondary antibodies using standard Western blotting procedures. Enhanced chemiluminescent substrate (FWPD02, Michigan Diagnostics, Royal Oak, MI, USA) was used to visualize bands.

The antisera/antibodies were used at the following dilution: Rabbit anti-Akt (pan) (C67E7) (1:1000; Cell Signaling Technology #4691), rabbit anti-Phospho-Akt (Ser473) (1:1000; Cell Signaling Technology #9271), mouse anti-GAPDH G-9 (1:1000; sc-365062, Santa Cruz Biotechnology, Dallas, TX, USA), goat anti-rabbit peroxidase conjugate (1:20,000; 111-005-003, Jackson ImmunoResearch Laboratories, West Grove, PA, USA), and goat anti-mouse peroxidase conjugate (1:20,000; Jackson ImmunoResearch Laboratories 115-035-166).

For quantification of protein levels, the intensity of pAkt and Akt bands was measured and normalized to GAPDH using Fiji (Version 2.18.0) [76]. To determine the relative amount of pAkt to total Akt, the value obtained for pAkt was divided by that obtained for Akt. Measurements were done with four Western blots using independent samples. Statistical analysis was done with GraphPad Prism using nonparametric Mann–Whitney tests.

## 5. Conclusions

Our results support the potential of WS as a therapeutic agent for promoting healthy aging and mitigating age-related declines in fitness. We found that the protective function of WS is mediated by reducing Akt phosphorylation and therefore its activity. Our genetic manipulations also show that at least three downstream pathways of Akt play a role in the effects of WS; the TSC/mTOR, FOXO, and the NRF2/Keap1 pathways.

## Data Availability

The original contributions presented in this study are included in the article/Supplementary Materials. Further inquiries can be directed to the corresponding author.

## Supplementary Materials

The following supporting information can be downloaded at: https://www.mdpi.com/article/10.3390/ijms27188314/s1.
