# Supplementary material for: Withania somnifera (Ashwagandha) Improves Fitness in Aged Drosophila by Reducing AKT Phosphorylation
Source: Int J Mol Sci. 2026 Sep 18;27(18):8314. doi: 10.3390/ijms27188314 (PMC13607971; doi:10.3390/ijms27188314)
Supplement: Supplementary file 1 [file ijms-27-08314-s001.zip › Table S1.pdf]

A

Sex × WSAq Treatment

Genotype	Female relative change (%)	Male relative change (%)	Difference in relative change, F-M (pp)	Sex × Genotype (normalized) significant?	Sex × Genotype (normalized) P
Control	+36.3	+12	+24.3	No	0.0683
Akt RNAi	-28.8	-67.9	+39.1	No	0.1729
Akt PHΔ	+2.0	-14.2	+16.2	No	0.6744
Akt	+44.9	+114.1	-69.1	Yes	0.0028
Pdk1 RNAi	+21.7	+58.5	-36.8	No	0.2556
Pdk1	+197.3	-13.7	+211	Yes	<0.0001
Pten	-41.2	-32.7	-8.6	No	0.6789
Pten RNAi	-28.4	-11.7	-16.7	No	0.3831
Pi3k92E RNAi	+14.5	-30.7	+45.2	No	0.4191
Pi3k92E	+6.5	+1.0	+5.4	No	0.7816
InR	+12.6	+34.6	-22	No	0.5266
InR RNAi	-19.5	+68.3	-87.8	Yes	<0.0001
Foxo	+0.8	+10.1	-9.3	No	0.5649
Foxo RNAi	-33.5	-54.4	+20.9	No	0.3214
Tsc1	+101.9	+47.2	+54.7	Yes	0.0486
Tsc1 RNAi	+32.7	+40.7	-8	No	0.8720
Tsc2	+12.6	+69.5	-56.9	No	0.3731
Tsc2 RNAi	-35.1	-7.6	-27.6	No	0.1609
CnC	+210.8	+139.2	+71.6	No	0.2639
CnC RNAi	-42.3	+2.5	-44.8	No	0.1888
Keap1	-3.8	+37	-40.9	No	0.3596
Keap1 RNAi	+33.6	-0.9	+34.4	No	0.9180

B **Sex × Genotype (Vehicle)**

Genotype vs sex-matched control (vehicle)	Female relative change (%)	Male relative change (%)	Difference in relative change, F-M (pp)	Sex × Genotype (normalized) significant?	Sex × Genotype (normalized) P
Akt RNAi	-59.6	-65.9	+6.3	No	0.5423
Akt PHΔ	-66.4	-16.5	-50.0	Yes	0.0024
Akt	+15.1	-61.9	+76.9	Yes	<0.0001
Pdk1 RNAi	-43.2	-77.2	+34.1	Yes	0.0116
Pdk1	-62.0	-49.8	-12.2	No	0.3676
Pten	-27.7	-34.3	+6.5	No	0.6472
Pten RNAi	-22.9	-32.2	+9.3	No	0.4718
Pi3k92E RNAi	-78.8	-86.1	+7.3	No	0.6104
Pi3k92E	-4.8	-28.3	+23.5	No	0.1335
InR	-2.4	-38.5	+36.1	Yes	0.0102
InR RNAi	+28.1	-58.5	+86.6	Yes	<0.0001
Foxo	+22.3	-30.4	+52.6	Yes	0.0001
Foxo RNAi	-21.2	-43.1	+21.9	No	0.1223
Tsc1	-44.5	-40.7	-3.8	No	0.7886
Tsc1 RNAi	-66.4	-77.2	+10.8	No	0.4897
Tsc2	-64.7	-84.8	+20.1	No	0.2595
Tsc2 RNAi	-18.2	-29.1	+10.8	No	0.4267
CnC	-87.3	-81.1	-6.2	No	0.5704
CnC RNAi	-57.9	-55.6	-2.3	No	0.8570
Keap1	-82.2	-76.5	-5.7	No	0.6111
Keap1 RNAi	0.0	+4.3	-4.3	No	0.7527

Table S1) Statistical analyses testing interactions between WSAq treatment and sex (A) and genetic background and sex in vehicle treated flies (B).
